# Dual Effects of Hydrogen Sulfide Donor on Meiosis and Cumulus Expansion of Porcine Cumulus-Oocyte Complexes

**DOI:** 10.1371/journal.pone.0099613

**Published:** 2014-07-01

**Authors:** Jan Nevoral, Jaroslav Petr, Armance Gelaude, Jean-Francois Bodart, Veronika Kucerova-Chrpova, Marketa Sedmikova, Tereza Krejcova, Tereza Kolbabova, Marketa Dvorakova, Alena Vyskocilova, Ivona Weingartova, Lenka Krivohlavkova, Tereza Zalmanova, Frantisek Jilek

**Affiliations:** 1 Department of Veterinary Sciences, Czech University of Life Sciences in Prague, Prague, Czech Republic; 2 Research Institute of Animal Production, Prague, Czech Republic; 3 Laboratoire de Régulation des Signaux de Division – EA 4479, Université Lille1, Sciences et Technologies, IFR 147, Villeneuved'Ascq Cedex, France; Institute of Zoology, Chinese Academy of Sciences, China

## Abstract

Hydrogen sulfide (H_2_S) has been revealed to be a signal molecule with second messenger action in the somatic cells of many tissues, including the reproductive tract. The aim of this study was to address how exogenous H_2_S acts on the meiotic maturation of porcine oocytes, including key maturation factors such as MPF and MAPK, and cumulus expansion intensity of cumulus-oocyte complexes. We observed that the H_2_S donor, Na_2_S, accelerated oocyte *in vitro* maturation in a dose-dependent manner, following an increase of MPF activity around germinal vesicle breakdown. Concurrently, the H_2_S donor affected cumulus expansion, monitored by hyaluronic acid production. Our results suggest that the H_2_S donor influences oocyte maturation and thus also participates in the regulation of cumulus expansion. The exogenous H_2_S donor apparently affects key signal pathways of oocyte maturation and cumulus expansion, resulting in faster oocyte maturation with little need of cumulus expansion.

## Introduction

Previously, molecules of some gases have been discovered to have biological activities. These gases, so called gasotransmitters, act as second messengers in the signal transduction of cell communication. In addition to the earlier observed nitric oxide and carbon monoxide, the role of hydrogen sulfide in cell metabolism has recently been studied [1]. Hydrogen sulfide (H_2_S) is enzymatically released from aminoacid L-cystein by Cystathionine β-Synthase (CBS), Cystathionine γ-Lyase (CSE) and 3-Mercaptopyruvate Sulfurtransferase (3-MPST) [2]–[4]. These enzymes are expressed in several tissues, including in the reproductive system [5]–[7], where it can be assumed that H_2_S production mediates physiological functions. The presence and effect of CBS in the ovarian follicles of mice has been determined [8], [9]. The role of H_2_S in oocyte maturation is not yet clear and has not been unravelled.

Successful meiotic maturation of oocytes is an important precondition of reproductive biotechnological progress. Only fully grown dictyate oocytes in germinal vesicle stage (GV-oocytes) undergo complete meiotic maturation and achieve metaphase II [10]. This process resumes after the hormonal stimuli action of the oocyte reinitiates meiotic division by the activation of key regulatory factors, such as Maturation/M-phase Promoting Factor (MPF) and Mitogen Activated Protein Kinase (MAPK), resulting in germinal vesicle breakdown (GVBD). Activation and correct kinesis of these factors are further necessary for meiosis I to II transition, organisation of the second meiotic metaphase spindle and spontaneous metaphase II-block [11]–[17]. The cytoplasmic changes of key factors of oocyte maturation are dependent upon intercellular communication between oocyte and surrounding cumulus cells [10]. On the other hand, mucification of the cumulus cells, known as cumulus expansion, causes a decrease of inhibitory substance flows into oocyte, especially cAMP, and restricted input of cAMP allows MPF activation, which triggers GVBD [18].

The cumulus expansion consists of synthesis and accumulation of glycosaminoglycans, especially hyaluronic acid, into the extracellular space [19]. Thus, cumulus expansion expressed by hyaluronic acid content may be a possible marker of successful GVBD, meiotic maturation and developmental competence acquisition in oocytes used for biotechnologies, i.e. *in vitro* fertilisation, transgenesis or cloning [20]–[23].

Meiotic maturation and cumulus expansion are simultaneously regulated by a complex network of several signal pathways including cAMP-PKA, Plk1-Cdc25-Cdc2, PI3K-Akt and Mos-MEK-MAPK [24]–[28]. Noticeably, the PI3K-Akt and cAMP-PKA pathways have been reported to be regulated by H_2_S during the cell cycle of somatic cells [29]–[32]. Full knowledge of the molecular mechanisms of oocyte maturation and H_2_S involvement in meiosis could improve the yield of successfully *in vitro* matured oocytes. We hypothesised that H_2_S plays a role in the regulation of meiotic oocyte maturation. The aim of this study was to evaluate the influence of the H_2_S donor on oocyte maturation, regulatory kinase activity in oocytes and the cumulus expansion intensity of porcine cumulus-oocyte complexes (COCs) cultivated *in vitro*.

For this purpose, we tested the influence of the exogenous H_2_S donor, Na_2_S, on oocyte maturation, developmental competence acquisition and cumulus expansion of COCs. Here, we report for the first that the H_2_S donor acts on oocytes to regulate cumulus expansion and progression through meiosis.

## Materials and Methods

### *In Vitro* Oocyte Cultivation with H_2_S Donor

Porcine ovaries were obtained from non-cycling gilts at the local slaughterhouse (Jatky Plzen a.s., Plzen, Czech Republic). Ovaries were transported to the laboratory in a saline solution (0.9% NaCl) at 39°C. Cumulus-oocyte complexes (COCs) were collected from ovarian follicles with a diameter of 2 – 5 mm by a 20-gauge aspirating needle. Only fully grown oocytes with intact cytoplasm surrounded by compact cumuli were used in further experiments.

The COCs were matured in a modified M199 medium (Sigma-Aldrich, USA) supplemented with 32.5 mM sodium bicarbonate, 2.75 mM calcium L-lactate, 0.025 mg/ml gentamicin, 6.3 mM HEPES, 13.5 IU eCG: 6.6 IU hCG/ml (P.G.600; Intervet, Holland) and 5% (v/v) fetal bovine serum (Sigma-Aldrich, USA). The culture medium contained 150, 300, 600 or 900 µM Na_2_S.9H_2_O (Sigma-Aldrich, USA), the H_2_S donor. The COCs were matured for 6–48 hs in 3.5 cm Petri dishes (Nunc) containing 3.0 ml of culture medium at 39°C in a mixture of 5.0% CO_2_ in air.

### Evaluation of Oocyte Meiotic Maturation

At the end of culture, the COCs were treated with 1 mg/ml bovine testicular hyaluronidase (Sigma-Aldrich, USA) dissolved in M199 medium and cumulus cells were separated from oocytes by repeated pipetting through a narrow glass pipette. The oocytes were subsequently mounted on microscope slides with vaseline, covered with a cover glass, and fixed in ethanol-acetic acid (3∶1 v/v) for at least 48 h. The oocytes were stained with 1.0% orcein in 50% aqueous-acetic acid and examined under a phase contrast microscope. Five groups of meiotic maturation stages were determined in accordance with the published criteria by Motlik *et* Fulka [33]: GV – germinal vesicle, LD – late diakinesis, MI - metaphase I, AITI – anaphase I to telophase I transition, MII – metaphase II.

### Histone H1 and Myelin Basic Protein Double Assay

The COCs were matured for 12 – 48 hs with the H_2_S donor. At each time interval during the culture, COCs were denuded and 10 oocytes per sample were collected. Assays were performed in accordance with the protocol of Kubelka *et al.* [34], with slight modifications. Briefly, the oocytes were washed four times in 0.01% polyvinyl alcohol in PBS, and transferred into 5 µl of buffer containing 40 mM 3-[n-morpholino] propanesulfonic acid pH 7.2, 20 mM para-nitrophenyl phosphate, 40 mM β-glycerolphosphate, 10 mM EGTA, 0.2 mM EDTA, 2 mM dithiothreitol, 0.2 mM Na_3_VO_4_, 2 mM benzamidine, 40 µg/ml leupeptin and 40 µg/ml aprotinin. Samples were immediately frozen and stored in Eppendorf tubes at −80°C until assays were performed. An assay of MPF and MAP kinase activity by their capacity to phosphorylate external substrates, specifically histone H1 (H1) and Myelin Basic Protein (MBP), was performed. The kinase reaction was initiated by addition of 5 µl of buffer consisting of 100 mM 3-[n-morpholino] propanesulfonic acid pH 7.2, 20 mM para-nitrophenyl phosphate, 40 mM β-glycerolphosphate, 20 mM MgCl_2_, 10 mM EGTA, 0.2 mM EDTA, 5 µM cAMP-dependent protein kinase inhibitor, 2 mM benzamidine, 40 µg/ml leupeptin, 40 µg/ml aprotinin, 600 µM ATP, 2 mg H1/ml, 3 mg MBP/ml) and 500 µCi/ml [γ-^32^P]ATP (GE Healthcare Life Sciences, UK). The reaction was conducted for 30 min at 30°C and terminated by the addition of 10 µl Laemmli sample buffer and boiling for 3 min. After electrophoresis on 15% SDS PAGE gels, it was stained with Coomasie Blue R250, destained overnight, dried and autoradiographed. Phosphorylated histone H1 and MBP signals were visualised by MultiGauge 2.0 software and related to metaphase I oocytes after 24 h cultivation, where we expected the peak of kinase activity [34].

### Oocytectomy and OOXs Cultivation

The COCs obtained using the above-detailed procedure were oocytectomised in accordance with Prochazka *et al.* [35]. Each COC was immobilised with a holding pipette. A glass needle was then introduced through the cumulus cells and the oocyte into the holding pipette, allowing the ooplasm to be sucked into the holding pipette. After withdrawal of the needle, the ooplasm, but not the zona pellucida, was aspirated into the holding pipette by a burst of a negative pressure. The technique was performed in a drop of culture medium covered by mineral oil in a Petri dish. A set of 25 oocytectomised complexes (OOXs) was prepared within 30 min and immediately placed into the culture. The further cultivation of OOXs took place under the already described conditions.

### Hyaluronic Acid Assay

Groups of 25 COCs or OOXs were cultured for 12–48 hs in 1 ml culture modified M199 medium. The culture medium with cumulus cells after denuding of oocytes, or with OOXs, was placed into an Eppendorf tube and centrifugated at 10 000 rpm for 10 min. Cell pellets were proteolytically digested by 30 µl Alcalase 2.4 L FG in PBS (1∶100 v/v, Novozymes, Denmark) for 2 hs and thereafter 30 µl Flavourzyme 1000 L (1∶100 v/v, Novozymes, Denmark) was added and the mixture was cultured for a further 3 hs. The reaction was terminated by boiling for 3 min and the samples were stored at −20°C until the assay was performed. In addition to cell pellet digestion, the aliquots of culture medium for hyaluronic acid measurement were prepared. The HA content was ascertained by enzyme-linked immunosorbent assay. The QnE Hyaluronic Acid ELISA Assay detection kit (Biotech, USA) was used to determine it. The amount of HA was measured spectrophotometrically on a microtitration plate using a Rainbow ELISA plate reader (wavelength 540 nm). The quadratic calibration curve was based on five standard concentrations of HA. Synthesis of HA was expressed either as the total HA production (HA content in cell pellet and medium) or the retained HA (HA content in cell pellet only). For each concentration of H_2_S donor and point of time scale, the measured values of total HA were related to the control group of oocytes after 48 h cultivation.

### Parthenogenetic Activation of Oocytes

Oocytes were partenogenetically activated using our previously published protocol [36]. Briefly, oocytes were matured *in vitro* for 44 and 46 hs with and without the H_2_S donor, respectively. After in vitro maturation, oocytes were denuded and activated for 5 min with 25 µM calcium ionophore A23185. After activation, the oocytes were cultured for 2 hs with 2 mM 6-dimethylaminopurine (DMAP) in NCSU23 medium [37]. The oocytes were then cultured for 24 hs or 7 days in four-well Petri dishes (Nunc) containing 1.0 ml of culture medium under described conditions. Subsequently, oocytes were fixed and stained as described above. Oocytes with pronuclei were considered to be activated. In a separate experiment after oocyte activation, the presumptive zygotes were cultured for 7 days. The cleavage rate and blastocyst achievement was assessed after 2 and 7 days of culture, respectively.

### Statistical Analysis

Our data are from at least three independent experiments. The general linear models (GLM) procedure in SAS software (SAS Institute Inc., USA) was used to analyse data from all experiments. Significant differences between groups were determined using the t-test. The level of significance was set at P<0.05.

## Results

### H_2_S Donor Accelerates Oocyte Maturation in a Dose-Dependent Manner

We evaluated the influence of different concentrations of H_2_S donor on the nuclear maturation of porcine oocytes after 20 and 30 hs of *in vitro* cultivation. Time points of 20 and 30 hs were selected to represent more meiotic stages.

**Figure 1:**
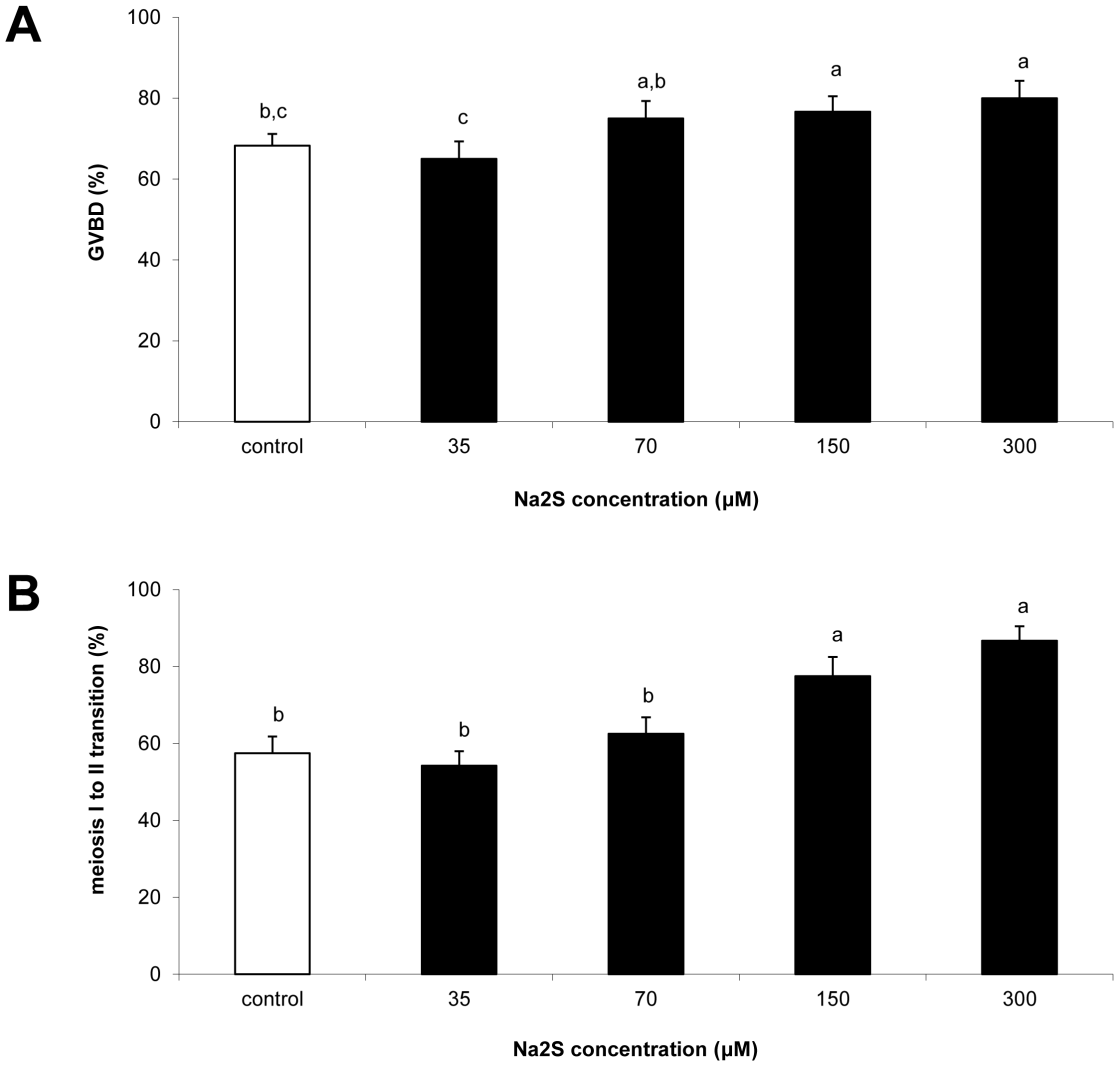
Effect of different Na_2_S concentrations on meiosis resumption and transition to meiosis II in oocytes. Proportion of GVBD (A) and meiosis I to II transition (B) during *in vitro* cultivation after 20 and 30 h *in vitro* cultivation, respectively. ^a,b,c^Statistically significant differences among experimental groups (P<0.05).

No effect of the H_2_S donor Na_2_S for the lowest concentration of 35 µM was observed after 20 and 30 h cultivation. With increasing concentration of Na_2_S accelerating GVBD (75.0–80.0 vs. 68.3% for H_2_S donor and control, respectively) after 20 h cultivation, the differences were statistically significant (Figure 1A, Table S1a). With higher concentration of the H_2_S donor, acceleration of meiosis I to II transition in oocytes was observed after 30 h cultivation (Figure 1B). As such, these oocytes achieved meiosis II with statistical differences in 77.5 and 86.7% of cases for 150 and 300 µM Na_2_S, respectively (see more in Table S1b).

### H_2_S Donor Accelerates Porcine Oocyte Maturation

**Figure 2:**
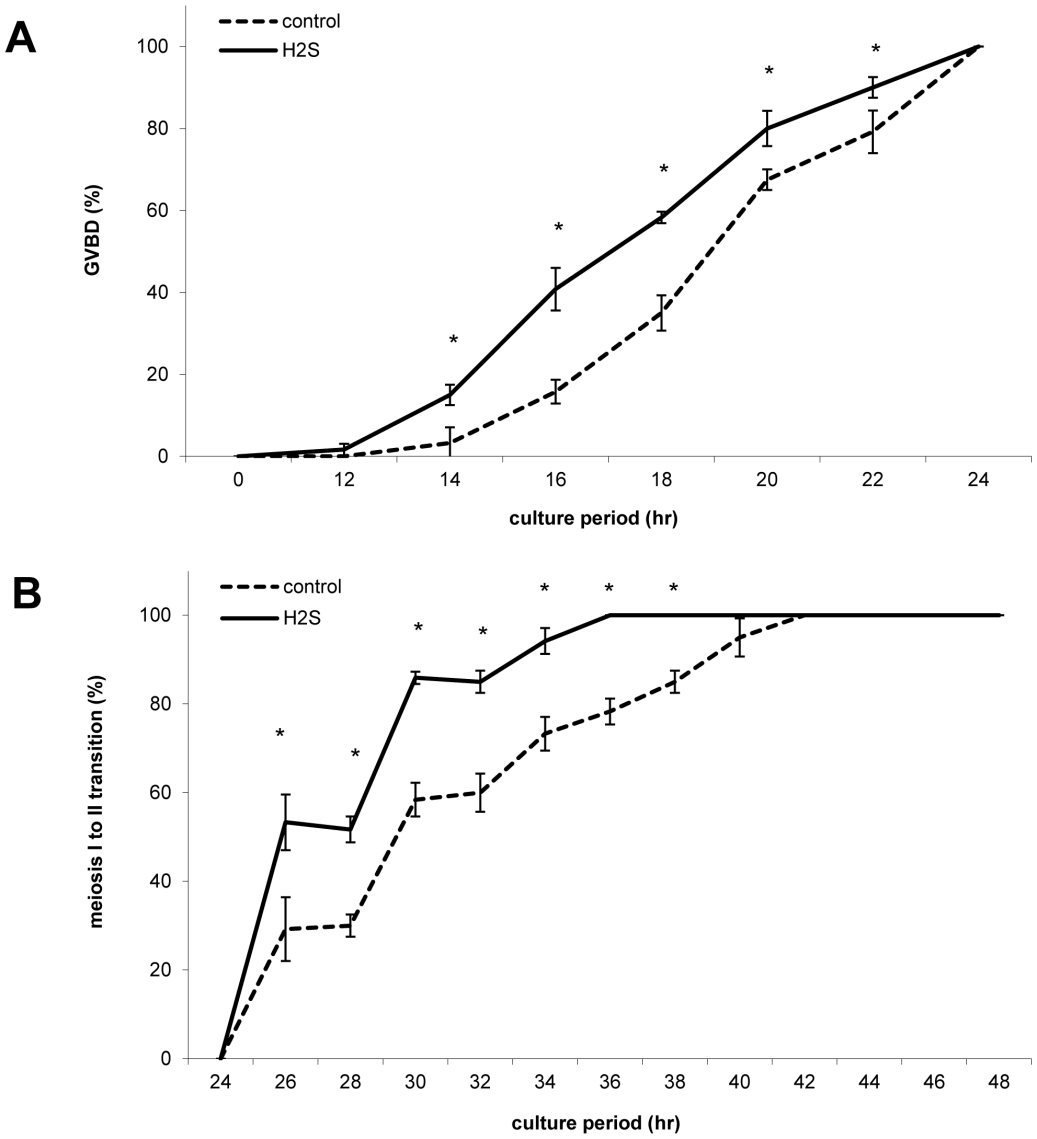
Effect of Na_2_S on meiotic resumption and transition to meiosis II during oocyte cultivation. Proportion of GVBD (A) and meiosis I to II transition (B) in oocytes during *in vitro* cultivation over 2 h time scale. H_2_S: 300 µM Na_2_S. *Statistically significant differences between control and H_2_S groups (P<0.05).

We evaluated the influence of H_2_S donor Na_2_S on nuclear maturation of porcine oocytes during *in vitro* cultivation over a 2 h time scale. We monitored the effect of 300 µM Na_2_S on germinal vesicle breakdown (GVBD). An accelerated decline of the amount of germinal vesicle (GV) oocyte together with GVBD increase were statistically significant after 14–20 h cultivation (Figure 2A). Moreover, H_2_S donor-treated oocytes reached faster meiosis II than the control ones (Figure 2B). The complete data are provided in Table S2.

### MPF and MAPK Activity Profiles Are Accelerated by H_2_S Donor

**Figure 3:**
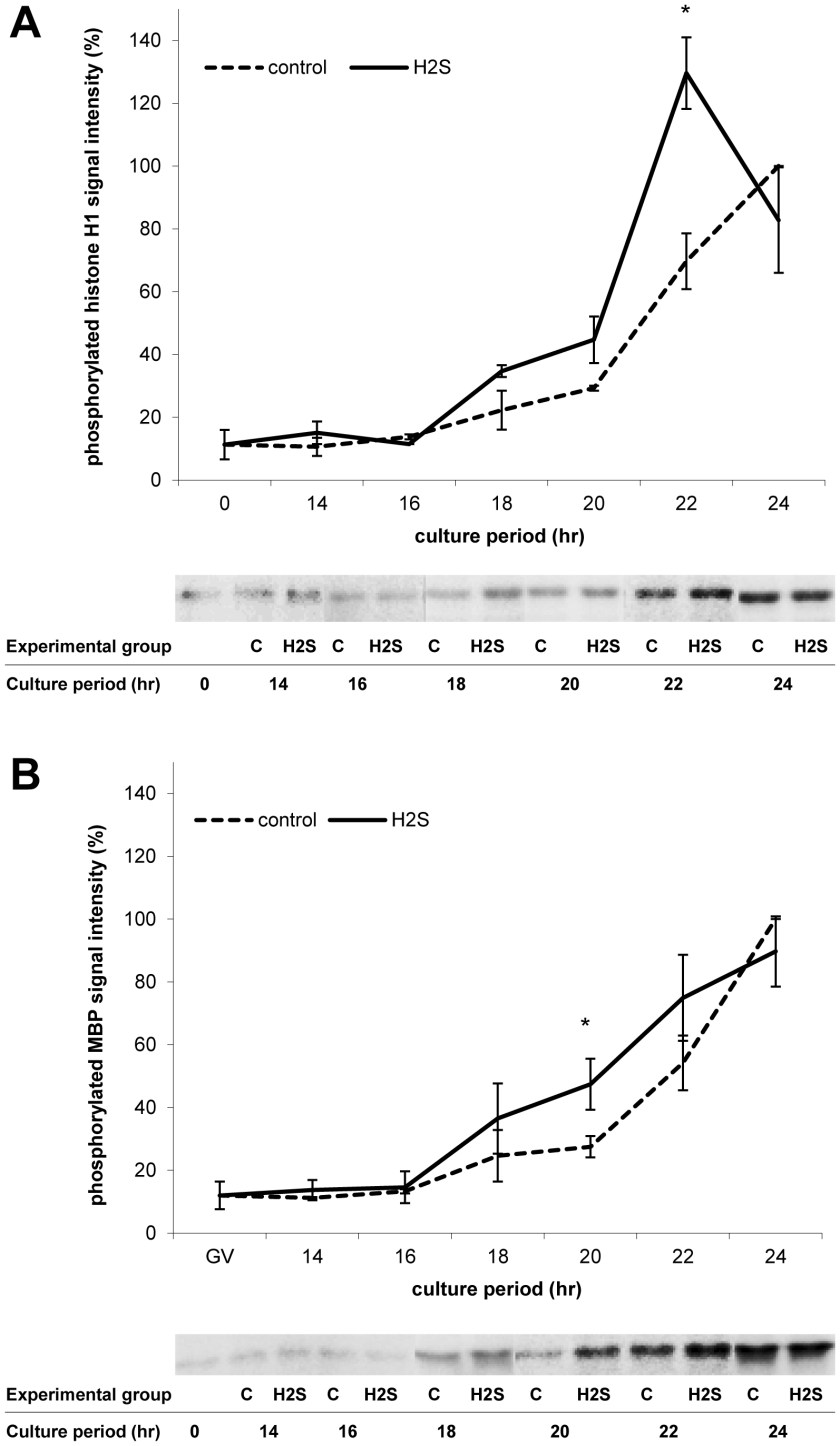
Effect of Na_2_S on MPF and MAPK activities during oocyte cultivation. Representative autoradiograms and signal quantifications of phosphorylated histone H1 (A) and MBP (B) reflecting MPF and MAPK activity, respectively. Kinase activity was measured in oocytes cultivated with or without Na_2_S over 2 h time scale. The kinase activity was related to oocytes cultivated for 24 hs. C: control; H_2_S: 300 µM Na_2_S. *Statistically significant differences between control and H_2_S groups (P<0.05).

To further characterise the effect of H_2_S on oocyte maturation, a kinase activity assay was performed (Figure 3A, 3B, Figure S1). We observed the influence of H_2_S donor, Na_2_S, in 300 µM concentration on the beginning of MPF and MAPK activity around GVBD over a 2 h time scale. Data were expressed relative to MPF/MAPK activity in oocytes cultivated for 24 h where it is predictable that kinase activity is the highest. The phosphorylated histone H1 and MBP signal intensities reflecting the MPF and MAPK activity profile, respectively, were increased and accelerated by the H_2_S donor during oocyte maturation. The difference in MAPK activity between the control and H_2_S groups was statistically significant after 20 h *in vitro* cultivation. During further *in vitro* maturation, significant acceleration of MPF occurred after 22 h cultivation.

### H_2_S Donor Can Substitute for the Absence of Cumulus Cells

**Figure 4:**
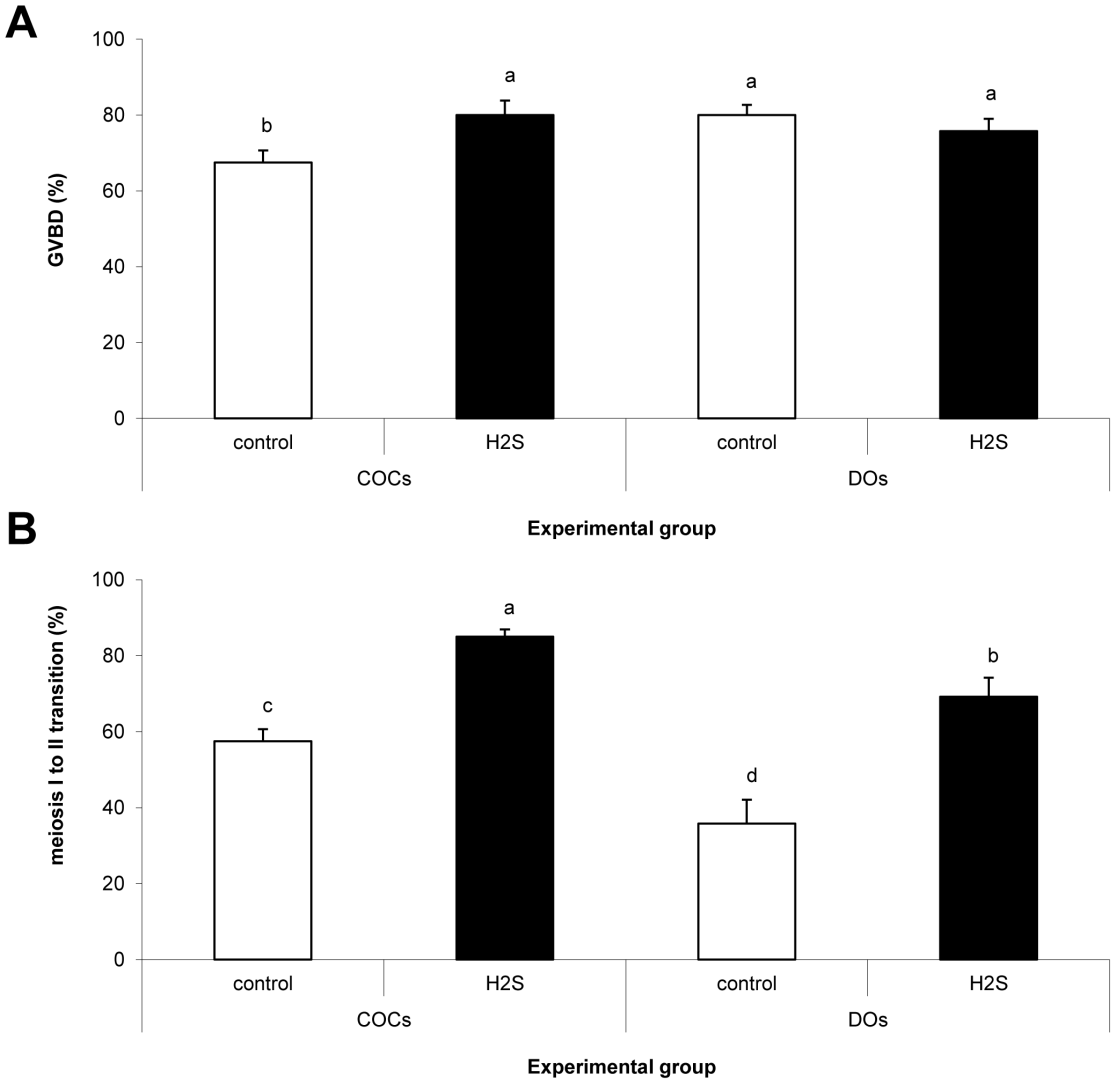
Effect of Na_2_S on meiosis resumption and transition to meiosis II in DOs. Proportion of GVBD (A) and meiosis I to II transition (B) during *in vitro* cultivation after 20 and 30 h *in vitro* cultivation, respectively. H_2_S: 300 µM Na_2_S. ^a,b,c^Statistically significant differences among experimental groups (P<0.05).

Denuded oocytes (DOs) were cultured with the H_2_S donor to evaluate cumulus cells' role during accelerated meiotic maturation. The aim of the experiment was to evaluate the GVBD and meiosis I to II transition of oocytes cultivated with 300 µM Na_2_S for 20 and 30 hs, respectively. No effect of Na_2_S on GVBD rates of DOs after 20 hs was observed. It should also be noted that in comparison to the control, more H_2_S-treated DOs reached nuclear stages of meiosis II after 30 hs (69.2 vs. 35.8% for H_2_S donor and control of DOs, respectively), see Figure 4. In addition, more DOs cultured with the H_2_S donor reached metaphase II (30.0%) in comparison with the control DOs and COCs (16.7 and 6.7%, respectively) and even COCs cultured with the H_2_S donor (15.8%). Further data are available in Table S3a and S3b.

### H_2_S Donor Influences Cumulus Expansion with Presence of Oocytes

**Figure 5:**
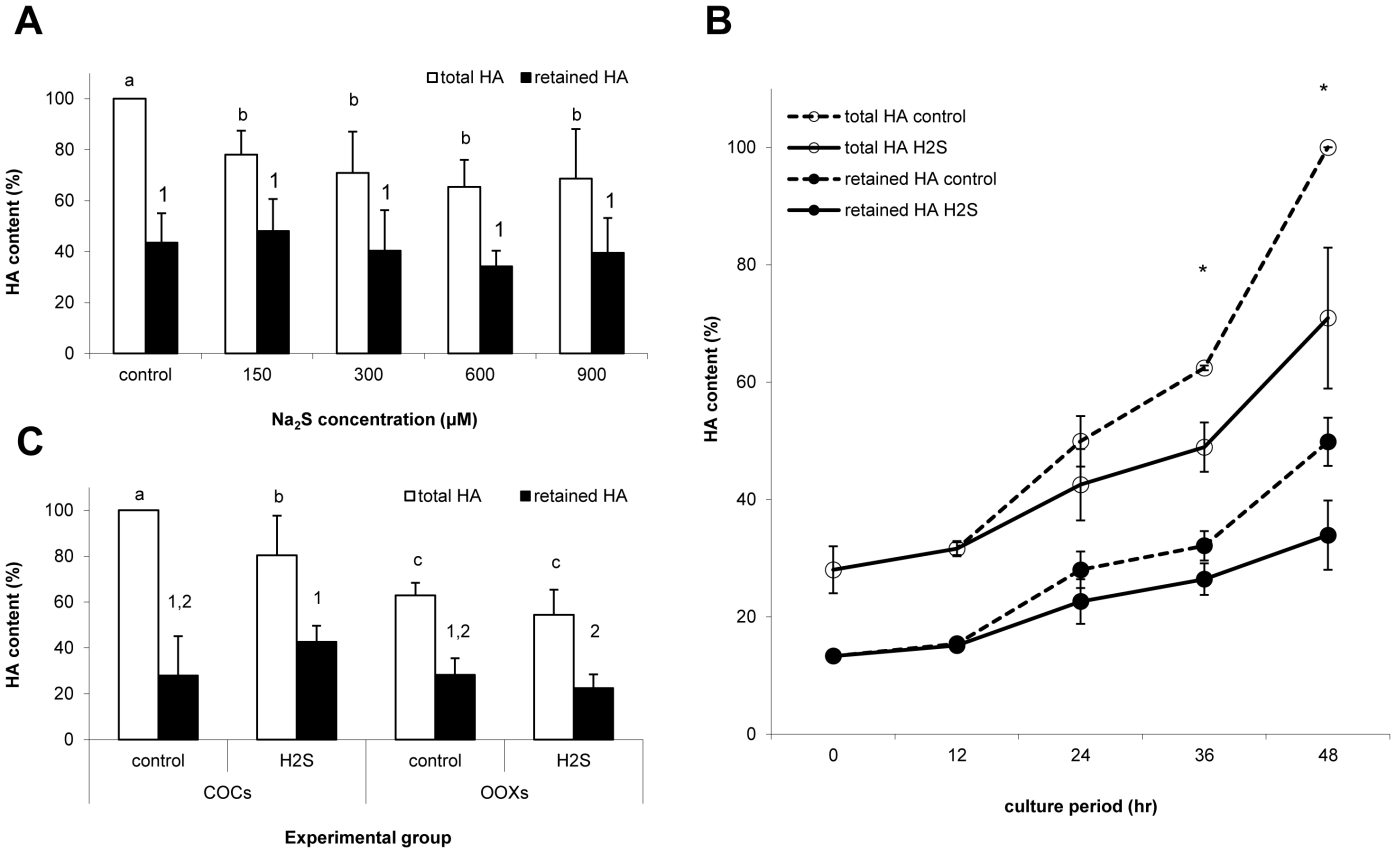
Effect of Na_2_S on HA content in expanded cumulus. (A) Total and retained HA content in COCs cultivated with 150–900 µM Na_2_S for 48 hs, total HA is related to the control group. (B) Total and retained HA content in COCs during *in vitro* cultivation with 300 µM Na_2_S over 12 h time scale, total HA is related to the control group after 48 h cultivation. (C) Total and retained HA content in COCs and OOXs cultivated with or without H_2_S donor, total HA is related to the control group of COCs. H_2_S: 300 µM Na_2_S. ^a,b,c^Statistically significant differences among experimental groups in total HA, ^1,2^statistically significant differences among experimental groups in retained HA, *statistically significant differences in total HA between control and H_2_S groups (P<0.05).

The aim of the experiment was to measure cumulus expansion by hyaluronic acid (HA) content in COCs and OOXs. The total HA production was assessed by HA content released into the culture medium and by retained HA in cell lysate. The total and retained HA was measured in COCs after 48 h *in vitro* cultivation and during maturation after 12, 24, 36 and 48 hs. The results are compared to control COCs after 48 h cultivation. It was observed that H_2_S donor, Na_2_S, inhibited total HA production after 48 hs by 21.9–34.6%. No dose-dependent manner was observed, differences are statistically significant (Figure 5A). For further experiments, a concentration of 300 µM Na_2_S was used.

HA production during *in vitro* cultivation of COCs is low after 12 hs of cultivation and it increased after 24 hs without significant differences between the control and H_2_S groups. The H_2_S donor significantly inhibited total HA production after 36 and 48 h cultivation by 13.0 and 29.0%, respectively (Figure 5B).

To evaluate the influence of oocyte presence on HA production and cumulus expansion, oocytectomised complexes (OOXs) were cultivated with the H_2_S donor for 48 hs. It was found that oocytectomisation reduced total HA in OOXs cultivated in a pure medium by 37.0%. HA production by OOXs cultivated with H_2_S donor decreased with no statistical significance in comparison with the above-mentioned OOXs. The data are shown in Figure 5C.

### H_2_S Donor Increases Activation Rate but It Has No Effect on Parthenogenetic Development

The influence of the H_2_S donor on developmental competence acquisition during *in vitro* oocyte maturation was examined. The oocytes were matured with 300 µM Na_2_S and in pure medium for 44 and 46 hs, respectively, when 100% of oocytes in both group were matured (see Table S2). The H_2_S donor in maturation medium significantly increased the activation rate (91.7 vs. 75.8% for H_2_S donor and control, respectively). The cleavage rate, morula and blastocyst formation were not influenced (Table 1).

**Table 1:** Effect of Na_2_S on partenogenetic development of porcine oocytes.

	Activation rate (24 hs)	n	Cleavage rate (2days)	Stage of early embryonic development (7 days)		n
				Morula	Blastocyst	
control	75.8±3.2	120	63.3±7.2	26.7±7.2	23.3±2.7	120
H_2_S	91.7±3.3*	120	70.8±5.0	30.8±1.7	25.0±4.3	120

Oocytes were matured with or without Na_2_S and partenogenetically activated using calcium ionophore. Pronucleus formation after 24 h zygote culture, cleavage rate after 2 days and blastocyst achievement after 7 days presumptive embryos culture were evaluated (%±SE).

H_2_S: 300 µM Na_2_S during oocyte maturation.

*Statistically significant differences between control and H_2_S group – in column (P<0.05).

## Discussion

In this study, we observed the relevant impact of the exogenously added H_2_S donor on porcine oocyte maturation. Originally, H_2_S was described as a toxic gas [38]. However, H_2_S is also endogenously generated in many types of mammalian cells, where it acts as a signal molecule, known as a gasotransmitter [2]. The concentrations of H_2_S donor we used are comparable to physiological values in tissues [2], [3] and we could assume that the observed effects of H_2_S donor exogenously added were not a result of its toxicity but rather relied on the physiological effect of H_2_S as a gasotransmitter. To the best of our knowledge, this study is the first one to describe the influence of the H_2_S donor on meiotic maturation of oocytes.

Significant acceleration of oocyte maturation during *in vitro* cultivation of porcine cumulus-oocyte complexes (COCs) with the H_2_S donor was observed. In agreement with a former study [34], meiotic maturation of oocytes was accelerated by an earlier increase of MPF and MAPK regulating oocyte maturation. The mechanisms underlying this precocious activation of MPF/MAPK induced by H_2_S remain to be determined. It is known that H_2_S can influence the activity of various factors including kinases by their direct sulfhydration [39], but no direct effect of H_2_S on MPF and MAPK activities has been yet reported. In addition to possible direct regulation, H_2_S may act indirectly on kinase activity by modifying other molecules, such as ion channels [40], and/or through regulation of up-stream kinases [30], [31]. Thus, the sulfhydration of these proteins may tune and control the oocyte maturation processes. In somatic cells, H_2_S-stimulation of signal pathways of cAMP/PKA [32] and PI3K/Akt [31] was observed. The important contribution these signal pathways make to kinase activity control during mammalian oocyte maturation is known [10], [41]. The experiments undertaken demonstrate that the H_2_S donor does not suppress acquisition of oocyte developmental competence during their *in vitro* maturation.

In our experiments, the action of the H_2_S donor on oocyte maturation in porcine COCs poses the question of whether the H_2_S donor effect is the result of direct function in oocytes, or whether the action of exogenous H_2_S is transduced by cumulus cells. Our results suggest that the H_2_S donor acts directly on the oocyte. Indeed, accelerated maturation by the H_2_S donor was observed in denuded oocytes (DOs) cultivated after removal of cumulus cells. The acceleration of meiotic maturation in H_2_S donor treated DOs was even more marked than in treated COCs. An explanation for this phenomenon could be in exogenous H_2_S retention in cumulus cells and/or in the extracellular matrix produced by these cells. This results in a smaller quantity of H_2_S being available for the oocytes. In addition, the H_2_S donor may cause processes inhibiting oocyte maturation in cumulus cells [42]. Accordingly, the immediate H_2_S donor influence induces faster meiotic maturation of DOs.

The influence of the H_2_S donor on cumulus cells was demonstrated by our subsequent experiments, in which we measured the level of hyaluronic acid (HA) in the extracellular matrix of cumulus cells as a marker of cumulus expansion. We showed inhibition of HA production in COCs cultivated with the H_2_S donor. The effect of the H_2_S donor on HA production was observed in all the concentrations of the H_2_S donor used after 48h *in vitro* cultivation. The H_2_S donor significantly influenced HA production in the second moiety of COC cultivation. A previous study had illustrated the decrease in activity of factors stimulating cumulus expansion, such as Cumulus Expansion Enabling Factor (CEEF), after metaphase I attainment [43]. We can presume that the role of the H_2_S donor may be in the deepening of CEEF decrease. The mechanism of H_2_S effect on cumulus expansion is as yet unclear. One possibility could be the influence of the above-mentioned cAMP/PKA signal pathway [32], which regulates cumulus expansion [44].

Cumulus expansion is extensively regulated by substances with oocyte origin [43], [44]. For this reason, we evaluated the influence of oocyte presence on cumulus expansion during cultivation with the H_2_S donor. We measured HA production in oocytectomied complexes (OOXs) where the oocyte had been removed. Our observation of decreased HA production after oocytectomy is in line with the previous study [45], where a decline of HA-synthase 2 expression in cumulus cells was shown. Whereas we demonstrated the inhibition of HA production in intact COCs cultivated with the H_2_S donor, no effect was observed in OOXs. It is known that production of CEEF by porcine cumulus cells is sufficient for cumulus expansion [35]. However, our experiments showed that inhibition of cumulus expansion by the H_2_S donor is mediated by the oocyte. Target systems in oocytes for H_2_S, regulating HA production in this way, remain unknown. Presumably, possible target molecules for exogenous H_2_S might be some members of the Transforming Growth Factor β superfamily which can be regulated by H_2_S [46] and subsequently influence HA-synthase 2 activity in cumulus cells [47].

The results of our study demonstrate that the H_2_S donor can participate in the regulation of oocyte maturation and cumulus expansion without the interference of developmental competence acquired during *in vitro* maturation. Further experiments are necessary for a full explanation of the role of H_2_S as a signal molecule and the mechanism of its effect during oocyte maturation, cumulus expansion and early embryogenesis.

## Supporting Information

Figure S1 **Effect of Na_2_S on kinase activity during oocyte cultivation.** Representative autoradiograms and signal quantifications of phosphorylated histone H1 (A) and MBP (B) reflecting MPF and MAPK activity, respectively. Kinase activity was measured in oocytes cultivated with or without Na_2_S in 6 hr time scale. The kinase activity was related to oocytes cultivated for 24 hrs. C: control; H_2_S: 300 µM Na_2_S. *Statistically significant differences between control and H_2_S group (P<0.05).
(TIF)

Table S1 **Effect of different Na_2_S concentrations on oocyte maturation after 20 hr (S1a) cultivation and 30 hr cultivation (S1b)**.
(DOC)

Table S2 **Effect of 300 µM Na_2_S on oocyte maturation.**
(DOC)

Table S3 **Effect of Na_2_S on maturation of DOs after 20 hr (S3a) cultivation and 30 hr cultivation (S3b)**.
(DOC)
