# Supplementary figures and images for: Dual Effects of Hydrogen Sulfide Donor on Meiosis and Cumulus Expansion of Porcine Cumulus-Oocyte Complexes
Source: PLoS One. 2014 Jul 1;9(7):e99613. doi: 10.1371/journal.pone.0099613 (PMC4077697; doi:10.1371/journal.pone.0099613)

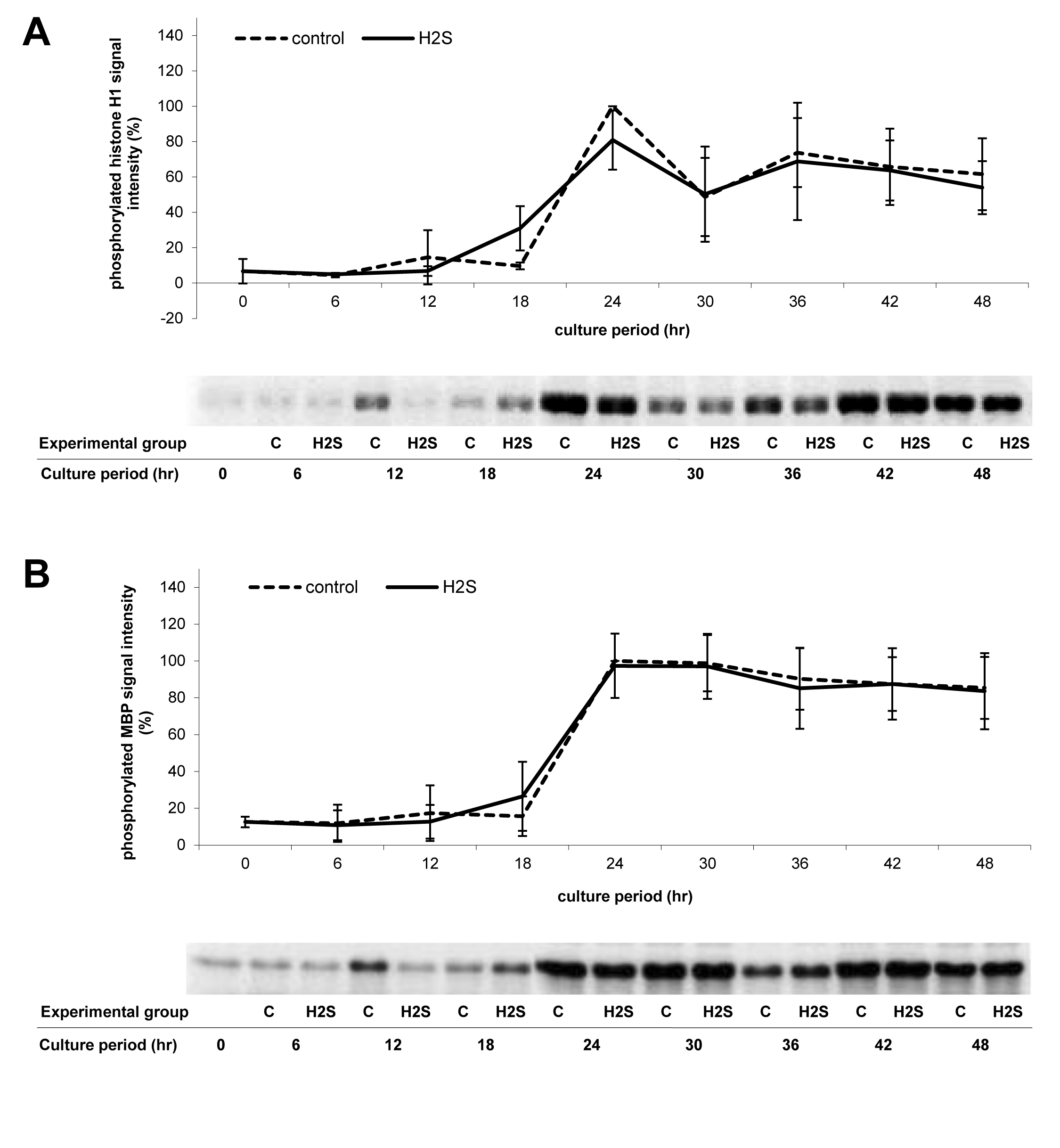

Supplement: Figure S1 — Effect of Na2S on kinase activity during oocyte cultivation. Representative autoradiograms and signal quantifications of phosphorylated histone H1 (A) and MBP (B) reflecting MPF and MAPK activity, respectively. Kinase activity was measured in oocytes cultivated with or without Na2S in 6 hr time scale. The kinase activity was related to oocytes cultivated for 24 hrs. C: control; H2S: 300 µM Na2S. *Statistically significant differences between control and H2S group (P<0.05). (TIF) [file pone.0099613.s001.tif]
