# Supplementary material for: Dual Effects of Hydrogen Sulfide Donor on Meiosis and Cumulus Expansion of Porcine Cumulus-Oocyte Complexes
Source: PLoS One. 2014 Jul 1;9(7):e99613. doi: 10.1371/journal.pone.0099613 (PMC4077697; doi:10.1371/journal.pone.0099613)
Supplement: Table S1 — Effect of different Na2S concentrations on oocyte maturation after 20 hr (S1a) cultivation and 30 hr cultivation (S1b). (DOC) [file pone.0099613.s002.doc]

# Supporting Information S1

Table S1a. Effect of different Na_2_S concentrations on oocyte maturation after 20 hr cultivation.

|  | Stage of meiotic maturation (% ± SE) | | | | | n |
| --- | --- | --- | --- | --- | --- | --- |
|  | GV | LD | MI | AI/TI | MII |
| control | 31.7±2.9^a,b^ | 25.0±2.5^a^ | 43.3±1.4^d^ | - | - | 120 |
| 35μl | 35.0±4.3^a^ | 25.0±4.3^a^ | 40.0±0.0^d^ | - | - | 120 |
| 70μl | 25.0±4.3^b,c^ | 20.0±5.0^a,b^ | 55.0±2.5^c^ | - | - | 120 |
| 150μl | 23.3±3.8^c^ | 16.7±1.4^b^ | 60.0±4.3^b^ | - | - | 120 |
| 300μl | 20.0±4.3^c^ | 7.5±2.5^c^ | 72.5±2.5^a^ | - | - | 120 |

GV: germinal vesicle oocytes; LD: late diakinesis ooccytes; MI: metaphase I oocytes; AI/TI: anaphase I to telophase I transition oocytes; MII: metaphase II oocytes. ^a,b,c,d^Statistically significant differences among experimental groups in the same nuclear stage – in column (P<0.05).

**Table S1b. Effect of different Na_2_S concentrations on oocyte maturation after 30 hr cultivation.**

|  | Stage of meiotic maturation (% ± SE) | | | | | n |
| --- | --- | --- | --- | --- | --- | --- |
|  | GV | LD | MI | AI/TI | MII |
| control | - | - | 42.5±4.3^a,b^ | 54.2±2.9^b^ | 3.3±1.4^c^ | 120 |
| 35μl | - | - | 45.8±3.8^a^ | 54.2±3.8^b^ | 0.0±0.0^c^ | 120 |
| 70μl | - | - | 37.5±4.3^b^ | 62.5±4.3^a^ | 0.0±0.0^c^ | 120 |
| 150μl | - | - | 22.5±5.0^c^ | 65.8±3.8^a^ | 11.7±1.4^b^ | 120 |
| 300μl | - | - | 13.3±3.8^d^ | 69.2±3.8^a^ | 17.5±6.6^a^ | 120 |

GV: germinal vesicle oocytes; LD: late diakinesis ooccytes; MI: metaphase I oocytes; AI/TI: anaphase I to telophase I transition oocytes; MII: metaphase II oocytes. ^a,b,c,d^Statistically significant differences among experimental groups in the same nuclear stage – in column (P<0.05).
