# Supplementary material for: Dual Effects of Hydrogen Sulfide Donor on Meiosis and Cumulus Expansion of Porcine Cumulus-Oocyte Complexes
Source: PLoS One. 2014 Jul 1;9(7):e99613. doi: 10.1371/journal.pone.0099613 (PMC4077697; doi:10.1371/journal.pone.0099613)
Supplement: Table S2 — Effect of 300 µM Na2S on oocyte maturation. (DOC) [file pone.0099613.s003.doc]

# Supporting Information S2

Table S2. Effect of 300μM Na_2_S on oocyte maturation.

|  |  | Stage of meiotic maturation (% ± SE) | | | | |  |
| --- | --- | --- | --- | --- | --- | --- | --- |
|  |  | GV | LD | MI | AI/TI | MII | n |
| 12hr | control | 100±0.0 | 0.0±0.0 | - | - | - | 120 |
|  | H_2_S | 98.3±1.4 | 1.7±1.4 | - | - | - | 120 |
| 14hr | control | 96.7±3,8 | 3.3±3.8 | 0.0±0.0 | - | - | 120 |
|  | H_2_S | 85.0±2.5^*^ | 0.0±0.0 | 15.0±2.5^*^ | - | - | 120 |
| 16hr | control | 84.2±2.9 | 0.0±0.0 | 15.8±2.9 | - | - | 120 |
|  | H_2_S | 59.2±5.2^*^ | 5.0±2.5^*^ | 35.8±5.2^*^ | - | - | 120 |
| 18hr | control | 65.0±4.3 | 10.8±1.4 | 24.2±2.9 | - | - | 120 |
|  | H_2_S | 41.7±1.4^*^ | 25.8±3.8^*^ | 32.5±5.0 | - | - | 120 |
| 20hr | control | 32.5±2.5 | 25.0±4.3 | 42.5±2.5 | - | - | 120 |
|  | H_2_S | 20.0±4.3^*^ | 6.7±2.9^*^ | 73.3±1.4^*^ | - | - | 120 |
| 22hr | control | 20.8±5.2 | 20.8±3.8 | 58.3±2.9 | - | - | 120 |
|  | H_2_S | 10.0±2.5^*^ | 15.0±2.5 | 75.0±2.5^*^ | - | - | 120 |
| 24hr | control | - | 3.3±3.8 | 96.7±3.8 | - | - | 120 |
|  | H_2_S | - | 0.0±0.0 | 100±0.0 | - | - | 120 |
| 26 h | control | - | - | 70.8±7.2 | 29.2±7.2 | - | 120 |
|  | H_2_S | - | - | 46.7±6.3^*^ | 53.3±6.3 | - | 120 |
| 28hr | control | - | - | 70.0±2.5 | 30.0±2.5 | - | 120 |
|  | H_2_S | - | - | 48.3±2.9^*^ | 51.7±2.9^*^ | - | 120 |
| 30hr | control | - | - | 41.7±3.8 | 54.2±5.2 | 4.2±1.4 | 120 |
|  | H_2_S | - | - | 14.2±1.4^*^ | 71.7±3.8^*^ | 14.2±2.9^*^ | 120 |
| 32hr | control | - | - | 40.0±4.3 | 50.0±5.0 | 10.0±2.5 | 120 |
|  | H_2_S | - | - | 15.0±2.5^*^ | 70.0±4.3^*^ | 15.0±2.5 | 120 |
| 34hr | control | - | - | 26.7±3.8 | 42.5±5.0 | 30.8±3.8 | 120 |
|  | H_2_S | - | - | 5.8±2.9^*^ | 56.7±2.9^*^ | 37.5±0.0^*^ | 120 |
| 36hr | control | - | - | 21.7±2.9 | 40.0±4.3 | 38.3±3.8 | 120 |
|  | H_2_S | - | - | 0.0±0.0^*^ | 37.5±6.6 | 62.5±6.6^*^ | 120 |
| 38hr | control | - | - | 15.0±2.5 | 34.2±3.8 | 50.8±1.4 | 120 |
|  | H_2_S | - | - | 0.0±0.0^*^ | 23.3±5.2^*^ | 76.7±5.2^*^ | 120 |
| 40hr | control | - | - | 5.0±4.3 | 35.8±1.4 | 59.2±3.8 | 120 |
|  | H_2_S | - | - | 0.0±0.0 | 8.3±1.4^*^ | 91.7±1.4^*^ | 120 |
| 42hr | control | - | - | - | 35.8±2.9 | 64.2±2.9 | 120 |
|  | H_2_S | - | - | - | 6.7±2.9^*^ | 93.3±2.9^*^ | 120 |
| 44hr | control | - | - | - | 23.3±1.4 | 76.7±1.4 | 120 |
|  | H_2_S | - | - | - | 0.0±0.0^*^ | 100±0.0^*^ | 120 |
| 46hr | control | - | - | - | - | 100±0.0 | 120 |
|  | H_2_S | - | - | - | - | 100±0.0 | 120 |
| 48hr | control | - | - | - | - | 100±0.0 | 120 |
|  | H_2_S | - | - | - | - | 100±0.0 | 120 |

H_2_S: 300μM Na_2_S. GV: germinal vesicle oocytes; LD: late diakinesis ooccytes; MI: metaphase I oocytes; AI/TI: anaphase I to telophase I transition oocytes; MII: metaphase II oocytes. ^*^Statistically significant difference between control and H_2_S in the same time point and the nuclear stage.
