# Supplementary material for: Dual Effects of Hydrogen Sulfide Donor on Meiosis and Cumulus Expansion of Porcine Cumulus-Oocyte Complexes
Source: PLoS One. 2014 Jul 1;9(7):e99613. doi: 10.1371/journal.pone.0099613 (PMC4077697; doi:10.1371/journal.pone.0099613)
Supplement: Table S3 — Effect of Na2S on maturation of DOs after 20 hr (S3a) cultivation and 30 hr cultivation (S3b). (DOC) [file pone.0099613.s004.doc]

# Supporting Information S4

**Table S4a. Effect of Na_2_S on maturation of DOs after 20 hrs.**

|  | | Stage of meiotic maturation (% ± SE) | | | | | n |
| --- | --- | --- | --- | --- | --- | --- | --- |
|  | | GV | LD | MI | AI/TI | MII |  |
| COCs | control | 32.5±3.2^a^ | 24.2±3.2^a^ | 43.3±2.7^c^ | 0.0±0.0^b^ | - | 120 |
|  | H_2_S | 20.0±3.8^b^ | 5.8±4.2^b^ | 74.2±3.2^a^ | 0.0±0.0^b^ | - | 120 |
| DOs | control | 20.0±2.7^b^ | 25.8±5.0^a^ | 54.2±3.2^b^ | 0.0±0.0^b^ | - | 120 |
|  | H_2_S | 24.2±3.2^b^ | 2.5±3.2^b^ | 38.3±4.3^c^ | 35.0±5.8^a^ | - | 120 |

H_2_S: 300μM Na_2_S. GV: germinal vesicle; LD: late diakinesis; MI: metaphase I; AI/TI: anaphase I to telophase I transition; MII: metaphase II. ^a,b,c^Statistically significant differences among experimental groups in the same nuclear stage – in column (P<0.05).

**Table S4b. Effect of Na_2_S on maturation of DOs after 30 hrs.**

|  | | Stage of meiotic maturation (% ± SE) | | | | | n |
| --- | --- | --- | --- | --- | --- | --- | --- |
|  | | GV | LD | MI | AI/TI | MII |  |
| COCs | control | 0.0±0.0^b^ | - | 42.5±3.2^b^ | 50.8±5.0^b^ | 6.7±2.7^c^ | 120 |
|  | H_2_S | 0.0±0.0^b^ | - | 15.0±1.9^d^ | 69.2±3.2^a^ | 15.8±4.2^b^ | 120 |
| DOs | control | 5.8±3.2^a^ | - | 58.3±4.3^a^ | 19.2±3.2^d^ | 16.7±4.7^b^ | 120 |
|  | H_2_S | 4.2±3.2^a^ | - | 26.7±2.7^c^ | 39.2±5.0^c^ | 30.0±5.4^a^ | 120 |

H_2_S: 300μM Na_2_S. GV: germinal vesicle; LD: late diakinesis; MI: metaphase I; AI/TI: anaphase I to telophase I transition; MII: metaphase II. ^a,b,c,d^Statistically significant differences among experimental groups in the same nuclear stage – in column (P<0.05).
